# Doses to the right coronary artery and the left anterior descending coronary artery and death from ischemic heart disease after breast cancer radiotherapy: a case-control study in a population-based cohort

**DOI:** 10.2340/1651-226X.2024.19677

**Published:** 2024-04-29

**Authors:** Mats Perman, Karl-Axel Johansson, Erik Holmberg, Per Karlsson

**Affiliations:** a Department of Oncology, Institute of Clinical Sciences, Sahlgrenska Academy, University of Gothenburg, Gothenburg, Sweden; b Department of Radiation Physics, Institute of Clinical Sciences, Sahlgrenska Academy, University of Gothenburg, Gothenburg, Sweden

**Keywords:** Breast cancer, cardiotoxicity, coronary arteries, late side-effects, cancer survivorship

## Abstract

**Background and purpose:**

Doses to the coronary arteries in breast cancer (BC) radiotherapy (RT) have been suggested to be a risk predictor of long-term cardiac toxicity after BC treatment. We investigated the dose–risk relationships between near maximum doses (D_max_) to the right coronary artery (RCA) and left anterior descending coronary artery (LAD) and ischemic heart disease (IHD) mortality after BC RT.

**Patients and methods:**

In a cohort of 2,813 women diagnosed with BC between 1958 and 1992 with a follow-up of at least 10 years, we identified 134 cases of death due to IHD 10–19 years after BC diagnosis. For each case, one control was selected within the cohort matched for age at diagnosis. 3D-volume and 3D-dose reconstructions were obtained from individual RT charts. We estimated the D_max_ to the RCA and the LAD and the mean heart dose (MHD). We performed conditional logistic regression analysis comparing piecewise spline transformation and simple linear modeling for best fit.

**Results:**

There was a linear dose–risk relationship for both the D_max_ to the RCA (odds ratio [OR]/Gray [Gy] 1.03 [1.01–1.05]) and the LAD (OR/Gy 1.04 [1.02–1.06]) in a multivariable model. For MHD there was a linear dose–risk relationship (1,14 OR/Gy [1.08–1.19]. For all relationships, simple linear modelling was superior to spline transformations.

**Interpretation:**

Doses to both the RCA and LAD are independent risk predictors of long-term cardiotoxicity after RT for BC In addition to the LAD, the RCA should be regarded as an organ at risk in RT planning.

## Introduction

Ionizing radiation to the heart increases the risk of late cardiac toxicity and mortality in a dose- and time-dependent manner [1]. Numerous studies have described the cardiotoxic effects from radiotherapy (RT) for breast cancer (BC) [2–4]. The risk of a major cardiac event increases linearly at 7.4% per Gray (Gy) of the estimated mean heart dose (MHD) [5]. In the meta-analysis published in 2005 by the Early Breast Cancer Trialist Group (EBCTG) including post-mastectomy RT trials from the 1970s and 1980s, an excess of non-breast-cancer mortality among irradiated patient is seen at 15 years of follow-up and increases up 20 years after RT. This excess is mainly explained by heart disease and lung cancer [6].

In RT after breast-conserving surgery and with the introduction of modern treatment techniques, long-term cardiac risks may no longer be clinically relevant for most patients [7, 8]. However, in a systematic review of heart doses reported in BC RT studies between 2003 and 2013, the average MHD was 5.4 Gy, indicating that long-term cardiotoxicity is still a relevant concern in modern BC RT [9].

Which anatomical substructures of the heart are the most important to protect in order to lower the risk of RT-related cardiotoxicity is not fully clear [10, 11]. Dose distribution studies have implicated radiation-related injury inflicted on the left anterior descending coronary artery (LAD) and left ventricle (LV) as underlying mechanisms of long-term cardiac morbidity and mortality. MHD constraints are used in RT dose-planning recommendations, and dose constraints to the LAD, in addition to LV have been suggested for the same purpose [12].

Several observations indicate that adjuvant RT for right-sided BC may increase the long-term risk of cardiac morbidity, though to a lesser magnitude than left-sided treatment [13, 14]. Various studies have been able to link patterns of stenosis in both the LAD and right coronary artery (RCA) to exposure to RT in a dose-dependent manner after BC RT [11, 15]. This relationship for RCA has also been described after RT for Hodgkin’s disease [16]. Dose distribution studies have shown that the RCA may receive significant doses even with modern RT techniques, especially if the lymph nodes of the internal mammary chain (IMC) are included in the target volume.

Any estimation of the excess risk of BC RT, right-sided or left-sided, compared to no RT will be affected by selection bias among patients treated after the cardiac risks of BC RT became known. BC patients in the modern era for whom RT is omitted are more likely at higher risk of cardiac events at baseline due to known risk factors for cardiac disease and other comorbidities [17].

The present study describes a cohort of long-term survivors after BC treated at our institution between 1958 and 1992. To the best of our knowledge, cardiac risk factors did not influence RT referral and RT decisions in the clinic during this period.

Our aim was to investigate the dose–risk relationships for exposure of both the RCA and LAD to RT and death due to ischemic heart disease (IHD) 10–20 years after BC diagnosis using individual dose estimations.

Our hypothesis was that both the RCA and LAD are at risk of radiation-induced pathology, and that exposure of any of these arteries to radiation independently contributes to the elevated risk of long-term cardiac toxicity after BC RT.

## Patients and Methods

### Population and data collection

**Figure 1:**
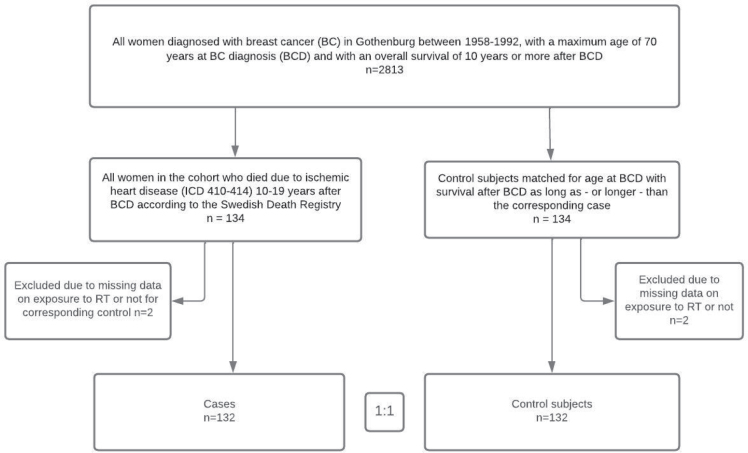
Cohort definition and selection of subjects for the case-control study.

Using the Swedish Cancer Registry, we identified all women who had been diagnosed with BC in Gothenburg between 1958 and 1992 and survived for 10 years or more. The age at breast cancer diagnosis (BCD) was set to a maximum 70 years. This yielded a cohort of 2,813 women. We used the Swedish Cause-of-Death Register to identify 134 cases of death in the cohort classified as due to IHD (ICD 410–414) during the period 10–19 years after BCD. Each case was matched to one control within the cohort based on age at BCD. All subjects in the cohort were eligible as controls if their survival time after BCD was at least as long as the corresponding case (Figure 1).

We searched individual patient records for cases and controls in The Sahlgrenska University Hospital Archives and the Regional Archives of Western Sweden for information on laterality, stage, surgery, and exposure to chemotherapy or endocrine therapy. Data were collected from patient charts while blinded to the patient’s status as a case or control. Every available RT treatment chart was reviewed. RT exposure was defined as left-sided, right-sided, or bilateral. We collected information on smoking, hypertension, diabetes, or pre-existing IHD at the time of BC diagnosis. Any connotation mentioning the existence of one of these potential modifying factors for cardiotoxicity was regarded as a positive finding.

### Radiotherapy reconstruction and estimation of absorbed doses

We used modern 3D-computer tomography (CT) planning scans with 5 mm between slices from 20 women with BC treated at our RT department (Sahlgrenska University Hospital). The CT scans were then divided into three categories based on heart volume: small, medium, and large. For each of the three categories, we chose the scans of the woman with distances from the heart to the jugular notch and sternum closest to the average distance. On the three selected CT scans, we delineated all volumes of interest. The RCA, the anterior descending branch of the LAD and the whole heart were defined as volumes of interest for this study. The coronary arteries were contoured by the same investigator (MP) in collaboration with one thoracic radiologist and one thoracic surgeon. The coronary arteries cannot be visualized in every slice; therefore, we used visible anatomical landmarks to create continuous volumes. Our definition of the LAD volume did not intend to include the left main common artery (LCA). We did not include all distal branches in the definition of the LAD or RCA and we did not define segments of the arteries. The volumes of the delineated coronary arteries were expanded with a 3D margin of 4 mm, and this expanded volume was used for dose evaluation. We also delineated the whole heart, the LV, and LCA for correlational analysis.

For each case and each control in the cohort treated with RT, we reconstructed the arrangement of treatment fields as stated in the individual’s chart. For this purpose, we used a treatment planning system on each of the three CT scans. The 3D dose distribution to the volumes of interest was calculated and dose–volume histograms (DVHs) generated. Each bin in the DVHs was converted by the linear quadratic model in order to obtain equivalent doses in 2 Gy fractions (EQD_2Gy/3_) with α/β = 3 Gy. From the DVH of the whole heart, we obtained the mean EQD_2Gy/3_ and from the DVHs of the two arteries, we obtained the near maximum EQD_2Gy/3_. The near maximum dose (D_max_) was defined in accordance with the ICRU to be the dose at 2% of the volume found in a cumulative DVH. The CT scan chosen from the medium heart volume category was used to estimate doses and modelling as stated in this report. The two CT scans chosen from the small heart volume and large heart volume groups were used for confirmatory analysis. The investigators who performed the reconstruction of RT and dose estimation were blinded to each patient’s status as case or control. See the Supplemental material for further details on RT reconstruction and dose estimation.

### Statistical analyses

Data were analyzed by univariable- and multivariable conditional logistic regression to assess risk factors and dose-response effects. Odds ratios (ORs) and 95% confidence intervals (CIs) were estimated. For exploratory reasons, we investigated dose–risk relationships for each heart volume of interest using piecewise spline transformation (cubic and linear), as well as simple linear modelling. Spline transformations are flexible models used to assess the effect of a continuous variable in a non-parametrical manner and to visually and/or statistically check the assumption of linearity in that effect [18]. A basic assumption is made for these transformations of piecewise relationships on an arbitrary number of segments. In this study, we segmented the material into quartiles depending on dose.

The likelihood-ratio test was used to test the superiority of fit and to compare the different models for each volume investigated. A two-sided *p*-value < 0.05 was considered significant. Stata software (version 16.1) was used for all statistical analyses.

### Ethical considerations

This study was approved by the Regional Ethical Committee (EPN) in Gothenburg (Dnr. S557-03, T025-05).

## Results

Information regarding exposure to RT was complete in 99% of all patients in the case-control study. For patients exposed to RT (*n* = 168), there was both written and graphical descriptions of treatment plans. Data at BC diagnosis and during the time at risk were complete for individuals in the case-control study on chemotherapy and endocrine treatment in 92 and 94%, respectively (Table 1).

**Table 1:** Demographics for cases and controls.

	Cases (*n* = 132)	Controls (*n* = 132)
Age at BCD, years (median, range)	62 (42–69)	62 (42–69)
Calendar year of BCD (median, range)	1968 (1958–1990)	1977 (1958–1992)
Interval between BCD and death of IHD, years (median, range)	14.9 (10.1–19.9)	
Type of surgery at BCD: - mastectomy - lumpectomy - none - missing data	124 4 – 4	119 9 1 3
Laterality of surgery: - right - left - bilateral - missing data	50 77 1 4	54 75 1 4
Chemotherapy exposure: - yes/no/missing	14/112/6	12/108/12
Tamoxifen exposure: - yes/no/missing	24/103/5	24/99/9
Potential modifying factors at BCD: - pre-existing IHD - smoking - diabetes - hypertension - any factor yes/no/missing	3 12 4 33 46/83/3	1 17 2 20 41/81/10
RT exposure: - RT confirmed - No RT	109 (83%) 23 (17%)	59 (45%) 73 (55%)

BCD: breast cancer diagnosis; IHD: ischemic heart disease; RT: radiotherapy.

RT exposure consisted of a variety of treatment techniques and regimens (Table 2).

**Table 2:** Characteristics of RT exposure for cases and controls.

	Cases exposed to RT *n* = 109		Controls exposed to RT *n* = 59	
	*n*	%	*n*	%
RT techniques: - Orthovoltage techniques - High energy techniques - Mixed	79 26 4	72 24 4	34 20 5	58 34 8
IMC in target - Yes - No	104 5	95 5	51 7	86 14
Laterality of RT - Right - Left Bilateral*	36 64 9	33 59 8	26 29 4	44 49 7
Prescribed dose per fx** - 2–2,9 Gy/fx - 3–3,9 Gy/fx - 4–4,9 Gy/fx - > 5 Gy/fx	8 78 19 4	7 72 17 4	8 32 14 5	14 54 17 8
EQD_2Gy/3_ Mean Heart Dose (P_25_–P_75_)	13,8 Gy (9,8–18,7)		12,8 Gy (7,0–17,4)	
EQD_2Gy/3_ Mean Dmax RCA (P_25_–P_75_)	28,4 Gy (24,5–31,6)		27,7 Gy (20,6–30,9)	
EQD_2Gy/3_ Mean Dmax LAD (P_25_–P_75_)	29,6 Gy (13,8–31,8)		20,7 Gy (11,6–31,4)	

* Laterality of RT defined as bilateral if any of the prescribed treatment fields included contralateral areas of the sternal midline on body surface projection.

** If various dose/per fx was prescribed in one treatment regimen, the lowest dose/fx value was chosen for categorization.

IMC: internal mammary chain; EQD: equivalent doses; RT: radiotherapy.

Treatment techniques and beam modalities changed considerably over the study period; orthovoltage treatment (OVT) was mainly used during 1958–1969 and megavoltage treatment (MVT) during 1970–1992. The target volume during the first period usually included IMC together with the chest wall. During the second period, the target volume and regimens varied.

The ORs and 95% CIs for IHD mortality were 8.0 (3.6–17.6) for RT vs. no RT, 6.2 (2.6–14.9) for right-sided RT vs. no RT, and 9.4 (4.1–21.8) for left-sided RT vs. no RT. The ORs for IHD mortality were 12.8 (5.1–32.2) and 4.5 (1.9–11.1) for exposure to OVT regimens and MVT regimens, respectively (Table 3).

**Table 3:** Odds ratios for IHD in univariate analysis.

**RT** No Yes **Treatment technique** High energy or mixed Orthovoltage **Laterality of RT** Right Left Bilateral	**OR (95%CI)** 1.0 8.0 (3.6–17.6) 4.5 (1.9–11.1) 12.8 (5.1–32.2) 6.2 (2.6–14.9) 9.4 (4.1–21.8) 13.4 (3.1–57.6)

IHD: ischemic heart disease; RT: radiotherapy.

**Figure 2:**
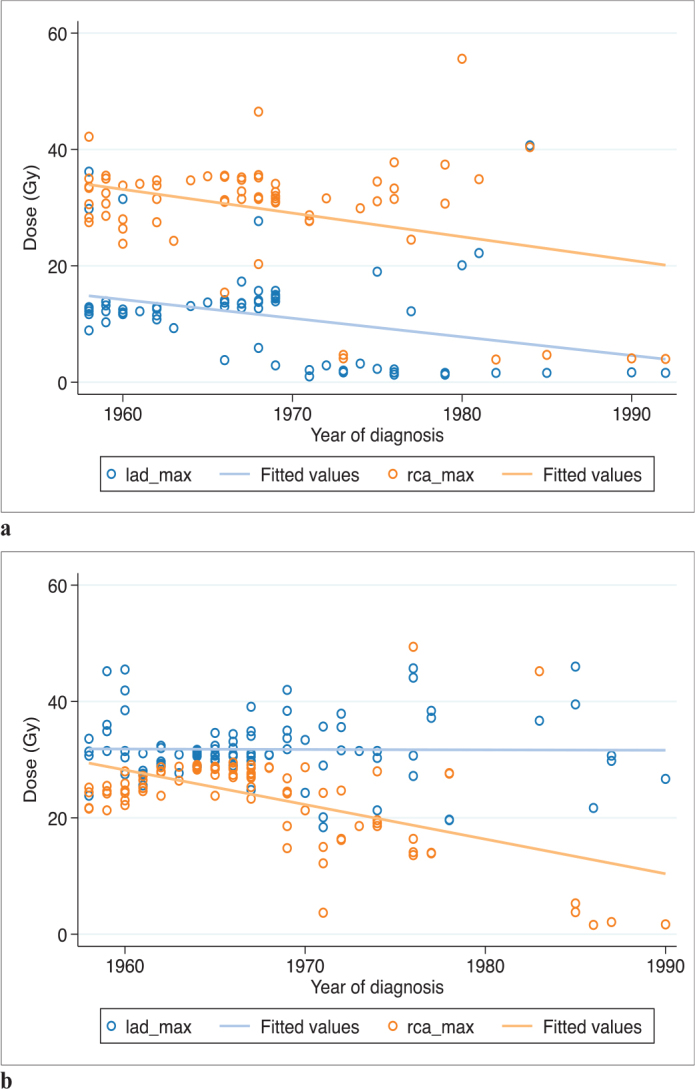
D_max_ to RCA and LAD over time. (a) Among right-sided exposed patients (*n* = 68). (b) Among left-sided exposed patients (*n* = 93). Patients defined as exposed to ‘bilateral treatment’ excluded (*n* = 13). RCA: right coronary artery; LAD: left anterior descending coronary artery.

The estimated EQD_2Gy/3_ to the two coronary arteries varied considerably among exposed women. Doses to the LAD among women with left-sided treatment did not vary significantly between 1958 and 1992. However, doses to both the RCA and LAD among women treated on the right side and doses to the RCA among women treated on the left side decreased with more modern treatment techniques (Figure 2).

**Figure 3:**
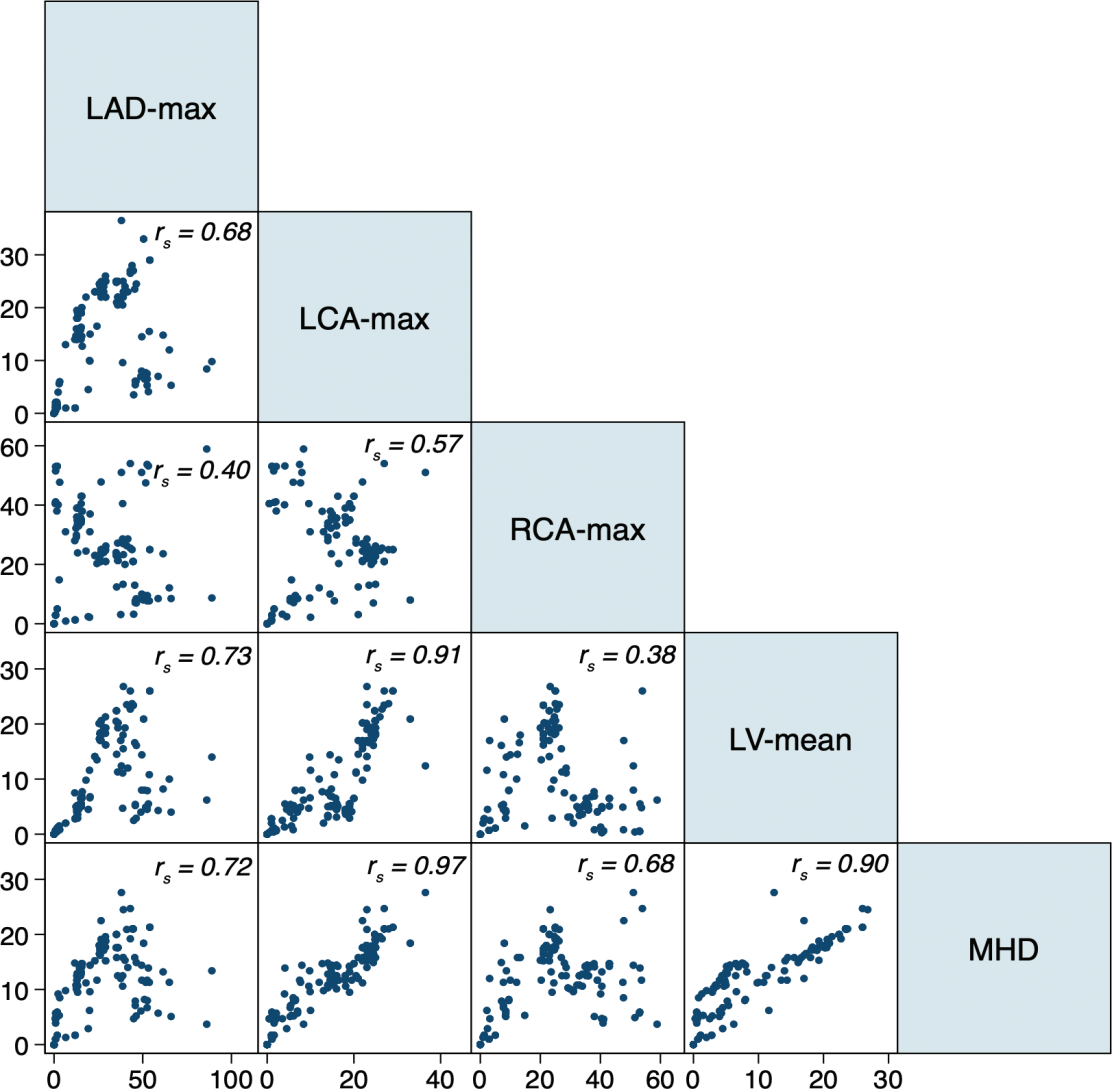
Correlation scatter plots for EQD_2_ adjusted near maximum doses (gray) to LAD (LAD-max), LCA (LCA-max), RCA (RCA-max), and mean dose to the left ventricle (LV-mean) and the whole heart (MHD) for all patients exposed to RT (*n* = 168). *r*_s_ = Spearman’s correlation coefficient. RCA: right coronary artery; LAD: left anterior descending coronary artery; LCA: left main common artery; EQD: equivalent doses; MHD: mean heart dose; RT: radiotherapy; LV: left ventricle.

Correlations between the D_max_ to the RCA and MHD and D_max_ to the LAD and MHD were high (*r*s = 0.68 and 0.72). The correlation between the D_max_ to the RCA and D_max_ to the LAD was weaker (*r*s = 0.40). LAD D_max_ strongly correlated with the D_mean_ to the LV (*r*s = 0.73), but the D_max_ to the RCA and D_mean_ to the LV were not (*r*s = 0.38; Figure 3).

**Figure 4:**
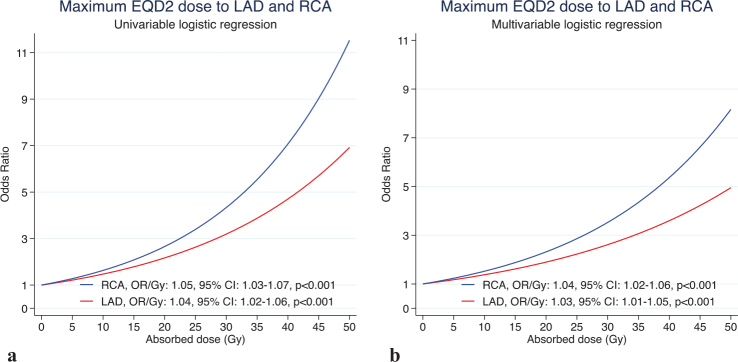
Dose–risk relationship between near maximum doses to the RCA and LAD and IHD in the (a) univariable linear logistic regression model and (b) multivariable logistic regression model. RCA: right coronary artery; LAD: left anterior descending coronary artery; EQD: equivalent doses; IHD: ischemic heart disease.

For the D_max_ to the RCA and LAD, flexible modelling (linear spline) showed a significantly superior fit in the univariate analysis compared to simple linear modelling. In the multivariate analysis including the D_max_ to both the RCA and LAD, there was no superiority of fit for flexible modelling over simple linear modelling. The multivariable linear model resulted in an independent increase in the OR with 1.04/Gy (95% CI 1.02–1.06) for the D_max_ to the RCA and an independent increase in the OR of 1.03/Gy (95% CI 1.01–1.05) for the D_max_ to the LAD (Figure 4).

For the MHD, univariate analysis did not show significant superiority of fit for flexible modelling compared with simple linear modelling. The linear model for the MHD resulted in an increased OR for cardiac mortality of 1.14/Gy (95% CI 1.08–1.19). We found almost identical results for the dose–response analyses of the LAD and RCA when RT reconstructions were performed on the CT scans chosen from the small and large heart groups (data not shown).

## Discussion

In this cohort treated with BC RT during 1958–1992 at our institution, we found linear relationships for the D_max_ to the RCA and LAD and the long-term risk of fatal IHD. We also confirmed a linear relationship between the MHD and the same endpoint.

Even if a risk association was found across different time periods and treatment techniques in the present study, the OVT including the IMC in the target volume had the greatest influence on risk. The first meta-analyses investigating non-cancer mortality in 10-year survivors of BC found that the excess risk of IHD was strongly influenced by the earliest RT trials applying treatment techniques similar to the OVT used at our institution during the 1950–1960s [3]. When comparing estimated doses to cardiac structures among exposed cases and controls in our study to data from other historical cohorts, the mean doses to the whole heart and near maximum doses to the LAD and RCA in Gothenburg clearly exceeded the estimates from other Scandinavian centers during the period 1958–1975 [19]. In our opinion, this is the main explanation for the estimated OR being 8.0 (3.6–17.6) for RT versus no RT in this cohort.

Our results regarding a dose–risk relationship for RCA are coherent with the findings of Nilsson et al. and Taylor et al., who were able to link patterns of stenosis in both the LAD and RCA to exposure to RT in a dose-dependent manner after BC RT, and Moignier et al., who found the same after RT for Hodgkin’s disease.

These findings add a piece of evidence supporting the assumption that both LAD and the RCA are structures that should be spared from high doses in RT if possible. Even if the RCA only receives scattered doses in most cases of modern BC RT [20], it should be considered an organ at risk, delineated as such, and doses should be kept at a minimum when relevant. This could be the case when optimizing RT treatment including the right IMC for a patient with known risk factors for long-term cardiac toxicity. Doses to the RCA should also be taken into account in RT planning for diagnoses other than BC, depending on dose levels (treatment-related) and cardiac risk factors (patient-related).

### Strengths and limitations

Follow-up in this study included two decades after BCD. Exposure to chemotherapy and endocrine was negligible, and we could not detect an uneven distribution of pre-existing IHD, smoking, hypertension, or diabetes among cases and controls. There were no findings in any of the patients’ charts on the treating physician’s reflection on heart disease or cardiological risk factors accompanying the decision on referring or not referring a woman to RT. We think that this observation indicates a low risk of confounding due to selection bias.

To estimate doses to the volumes of interest, we reconstructed the RT from individual charts based on data on surface anatomy and field size from drawn descriptions or photographs. This method is associated with several uncertainties. As described by Taylor et al., individual patient anatomy is the greatest source of variability [21]. We also performed our reconstruction on each of the other two CT scans from the small and large heart volume groups, and this did not change the results.

The use of non-contrast-enhanced CT scans with cardiac anatomical landmarks as a proxy for true artery location, as well as adding a margin of 4 mm to expand the volume of delineated coronary arteries, could be criticized. However, there were no established methods of cardiac segment delineation at the time we performed our RT reconstruction. In the cardiac contouring atlas for radiotherapy published by Duane et al. in 2017 [22], the authors have used the same anatomical landmarks that we had to rely upon for coronary artery delineation, consisting of the atrio- and interventricular grooves. We have no reasons to believe that there are significant differences in the estimation of coronary artery location between our study and the works of other groups. Individual variations in anatomy and motion artifacts are probably greater challenges methodologically.

There was a wide range of different doses and dose distributions to the volumes of interest among the exposed patients in this study. This lowers the risk of misinterpretation due to multicollinearity, that is a high degree of correlation in dose–volume parameters among various volumes-of-interest for one outcome.

We chose near maximum doses to the coronary arteries as variables in the regression model, considering the coronary artery as a ‘serial-like’ structure. From this perspective, high doses to limited parts of an artery are able to induce pathophysiological changes that compromise the function of the whole artery, which is to deliver oxygenated blood to that part of the myocardium. We further assumed that pathophysiological changes to one of these coronary arteries are sufficient for the induction of IHD.

In a cohort of 910 patients treated with RT after breast-conserving surgery between 2005 and 2007, the group of Crijns and collaborators found that dose to the LV was a better predictor than MHD for the excess risk of an acute coronary event within 9 years of follow-up [23].

The RCA doses in this study were not strongly correlated with the LAD and LV doses, further supporting the hypothesis of RT-induced stenosis to the RCA as a plausible explanation for the dose–risk relationship seen for the D_max_ to the RCA. For LAD doses and LV doses, correlations were stronger, and we cannot rule out that our estimation of the true absorbed dose to the coronary arteries to some extent also reflects the dose absorption by the underlying myocardium of the LV, especially for the older non-tangential techniques in this study.

Controls in the cohort were matched to case subjects by age at BCD but we did not match for calendar year of BCD in order to get as much variability in exposure as possible. As a result, the median year of BCD among cases was in median 9 years before the controls whereas the controls were equally distributed over the whole time period. We consider this difference in calendar year of BCD between cases and controls to be the result of the increased cardiotoxic effects of the OVT regimens applied before 1967, but this time difference is also a potential source of bias. We cannot rule out that prevention and treatment of IHD improved substantially for each decade between 1958 and 1992. Studies on IHD incidence and mortality in Gothenburg from this period show that a majority of patients who died of IHD died outside hospital and were unaware they had coronary heart disease before death. The same studies showed no clear overall trends in IHD mortality among women in Gothenburg between 1970 and 1985 [24, 25]. Based on these observations, we believe that the 9-year difference in median year of BCD between cases and controls does not bias our results.

In 2002, the percentage of erroneous classification regarding the three first digits of the ICD-codes in the SCDR was estimated to be 3.3 ± 0.4%. The classifications of cause-of-death in our population were mostly performed in the 70s, 80s, and 90s when the autopsy rate was much higher than today. The major uncertainty in cause-of-death coding is the clinical judgment of the reporting physician regarding the events leading to fatal outcome. The greater percentage of autopsies during the period when the majority of patients in this cohort died indicate a level of accuracy comparable with that seen in 2002. IHD mortality did not seem to represent a surrogate for BC death, as 93% of the cases died with no record of recurrent BC or new malignancy.

## Conclusion

In this population-based cohort of BC patients, we found that the dose to both the RCA and LAD predicted long-term cardiotoxicity. The dose–risk relationship that exists for MHD and late cardiac toxicity may be explained, in part by radiation-induced coronary artery disease. In addition to the LAD, the RCA should be considered an independent organ at risk in BC RT planning.

## Supplementary Material

Doses to the right coronary artery and the left anterior descending coronary artery and death from ischemic heart disease after breast cancer radiotherapy: a case-control study in a population-based cohort

## Data Availability

The authors state that the data supporting the article are available at the institution and may be shared at request as far as ethical and legal security aspects are followed.
