# Supplementary material for: Doses to the right coronary artery and the left anterior descending coronary artery and death from ischemic heart disease after breast cancer radiotherapy: a case-control study in a population-based cohort
Source: Acta Oncol. 2024 Apr 29;63:19677. doi: 10.2340/1651-226X.2024.19677 (PMC11332482; doi:10.2340/1651-226X.2024.19677)
Supplement: Doses to the right coronary artery and the left anterior descending coronary artery and death from ischemic heart disease after breast cancer radiotherapy: a case-control study in a population-based cohort [file AO-63-19677-s1.pdf]

Supplementary material has been published as submitted. It has not been copyedited or typeset by Acta Oncologica.

Supplementary material

Treatment reconstruction and estimation of dose

For treatment reconstruction, 20 representative non-contrast-enhanced 3D-CT planning scans were selected from treated BC patients at our RT department after the year 2005. All of these patients had undergone mastectomy and their mean age was the same as the mean age of the women in the present study. The patients were imaged in a supine position with CT slice thickness of 5 mm and pixel matrix of 512×512. The CT scans had been performed without any form of gating procedure. One of the investigators (MP) delineated the heart contour on all scans, and we estimated the heart location relative to the jugular notch and sternum. The total heart volume was defined by contouring the visible myocardium and pericardium, including the most proximal parts to the myocardium of the vena cava superior, pulmonary trunk and ascending aorta apically, and the lowest part of the myocardium/pericardium caudally. The CT scans were then divided into three categories based on heart volume: small (S), medium (M), and large (L). For each of the three categories, we chose the scans of the woman with distances from the heart to the jugular notch and sternum closest to the average distance. The intact breast on the contralateral side of the three CT sets were digitally removed on each CT slice based on the shape on the side of mastectomy in order to use the selected CT sets for all women independent of treatment side. The three selected full 3D-CT sets were then used for treatment reconstruction and definition of volumes of interest.

The volumes of interest were contoured on every slice for each of the three CT sets to create a full set of 3D volumes (Table S1). The location of the heart in relation to the midline of each CT set in a central CT slice is shown in Figure S1. We used the variation in volume and location

between the different CT sets to estimate the uncertainties of the dose distributions and for validation purposes in our dose response analyses.

Treatment regimens and techniques

All available information on RT exposure was collected from each patient´s chart. The charts commonly included a photograph or a drawing of the treatment fields. The graphical treatment description was absent for only one patient and information on RT exposure deduced from the medical notes. None of the 168 exposed women had undergone 3D-CT-based treatment planning, and only 13 patients had a single central plane contour at the chest wall before treatment planning. A summary of the treatment techniques and prescribed doses used to irradiate the internal mammary chain (IMC), chest wall, and breast are presented in Table S2. Orthovoltage treatment (OVT) was used for 113 subjects (67%), megavoltage treatment (MVT) for 47 subjects (28%), and a combination of OVT and MVT for 8 subjects (5%). Treatment techniques and beam modalities changed considerably over the study time period; OVT was mainly used during 1958-1969 and MVT during 1970-1992. The target volume during the first period usually included IMC together with the chest wall. The field arrangements for the OVT regimens exhibited little variation. Typically, one field perpendicular to the IMC was used with field sizes in the range of 4-7 cm × 10-16 cm and two fields with different energies, perpendicular to the thorax wall (tilted about 20 degrees). These two fields were divided into a cranial and caudal position. The border of the IMC field was often placed 1-2 cm on the contralateral side. The supraclavicular fossa and top of the axilla were treated with a separate field. The prescribed exposure per fraction during the first period was a standardized 300 Roentgen (R) units, to a total exposure of 2100 R to 3300 R.

During the second period, target volumes and regimens varied. The IMC (ipsilateral or bilateral) was treated with a 12 MeV electron beam (29 patients) and/or the chest wall with an 8 MeV

electron beam (9 patients). The breast/chest wall of 13 subjects was treated with tangential MVT photon beams. For the remaining women (64 patients), other combinations of beam energies, target volumes, and techniques were used. A considerable variation in the prescribed dose and accordingly, the dose per fraction was also observed during the second period, (Table S2).

Treatment planning system

The Eclipse® treatment planning system (TPS) was already configured for dose calculation of electron beams (8 to 15 MeV) and photon beams (^{60}Co, 4 to 6 MV). However, a configuration had to be performed for the two beam qualities used for recalculation of OVT; 200 kV 0.5 mm Cu filter and 140 kV 4mm Al filter. Dose reconstruction and dose calculation in a commercial TPS was not available from the TPS company for these beam qualities. For these purposes, we configured the TPS for a 3D dose calculation of OVT beams. To the best of our knowledge, no such configuration had been performed for any OVT previously. To configure the TPS for the OVT beam qualities previously in use at our department, we used stored isodose diagrams. Measurements could not be performed because the units had been removed. Five dose profiles and central depth dose distributions for five field sizes for the two beam qualities were extracted from the isodose diagrams, and these profiles were then loaded into the TPS. Based on the new configuration, the calculated dose distributions for five field sizes were then compared with the different old isodose diagrams. To improve the agreement, the profiles and kernels in the TPS had to be adjusted using a trial-and-error method. The agreement at different depth and SSD with the old printed isodose diagrams after the optimization procedure was better than 3 % and 3 mm in a homogeneous phantom. The 3D dose distributions were calculated in the TPS using a pencil beam convolution method with a modified Batho heterogeneity correction for tissue inhomogeneity.

Treatment reconstruction, estimation of dose, and generation of DVHs

We performed individual 3D dose reconstruction for the 168 patients on the three selected CT sets (S, M, and L). The dose calculation voxel size was 5×5×5 mm, and the dose bins in the histograms were 0.5 Gy. All information on the treatment regimens was used when reconstructing the field arrangements to the IMC, chest wall/breast, and other locations of interest. Only a few women had identical combinations of field configurations, prescribed doses, and beam qualities; therefore, all treatments had to be individually reconstructed. Due to the "low" heart dose contribution by the treatments of the axilla and/or supraclavicular fossa, they were excluded from the reconstruction [2]. In contrast, two women who had been exposed to palliative RT of the spinal vertebrae during the study period were included in the dose reconstruction because treatment contributed to the heart dose. To further improve dose reconstruction accuracy, interviews were made with radiation oncologists, medical physicist and nurses who were involved in OVT and MVT at our department from 1958 and onwards. The treatment plans were defined as the sum of the dose of all fields that contributed to the organs of interest. The OVT were prescribed in the unit Roentgen (R) and this exposure had to be converted to an equivalent prescription in Gy before calculation of the TPS. The absorbed dose of the OVT was then corrected by a factor of 1.18 to account for the enhanced biologic effectiveness [3]. For each of the 504 plans (168 patients with three different CT sets each) and the five volumes of interest, differential dose volume histograms (DVHs) were generated, and these 2520 DVHs were transferred to another computer for further manipulation. The DVHs were converted with the linear quadratic (LQ) model in order to obtain DVHs of the equivalent dose in 2 Gy fractions ($EQD_{2Gy/3}$). We assumed an α/β of 3 Gy as representative of late responding tissue. However, the differences obtained by using an α/β of 2 or 4 Gy turned out to be negligible compared to other uncertainties. The fractionation per field was used in the

conversion but, where the treatments had been given every second day, the overlapping volume had to be corrected based on the combined fractionation of the involved fields. From the DVHs of the five volumes, the mean $EQD_{2Gy/3,}$ the near maximum dose (D_{max}) $EQD_{2Gy/3,}$ and a number of cut-off values were obtained. The ICRU has defined the D_{max} to be the dose at 2% of the volume found in a cumulative DVH [1]. This measure replaces the maximum dose found in one single voxel, and we postulated this to be more accurate than the voxel maximum dose when relating exposure to risk.

DVHs

In Figure S2 four cumulative DVHs are presented with right- and left-sided treatments by two typical treatment techniques. The OVTs have large mean and D_{max} to the five volumes of specific interest. The dose distribution is relatively homogeneous, especially for the left-sided treatments. In contrast, the regimens given with the MVT technique result in much lower mean doses to the volumes of interest, but the near maximum doses are high for left-sided treatment of the LAD and heart. The reason for this is that parts of these volumes are located close to the tumor target.

Dose parameters

Investigation of the dose distribution resulted in a large variation in the $EQD_{2Gy/3}$ for the five volumes of interest, 168 treated patients, and the three CT sets (Table S3). A detailed presentation of the estimated mean and near maximum D_{max} of the five structures and three CT sets of all 168 patients is given in Tables S3-S4 and Figure S3. Some general results can be drawn. Due to their critical location in the anterior part of the heart, the mean and D_{max} for the LAD and RCA varied more than for other structures. A large difference in the mean and D_{max} LAD was also found for RCA doses between left- and right-sided treatments. The dose in the

heart, as expected, was lower for right-sided treatments than for left-sided treatments due to the heart´s position slightly to the left in the body. The IMC fields given with OVT were the main dose contributors to the five volumes (data not shown).

We found that the mean $EQD_{2Gy/3}$ was commonly a factor 2 to 3 lower (3 to 13 Gy) in the second time period (1970-92) than the first time period (1958-69). The mean $EQD_{2Gy/3}$ was 40-55% of the D_{max} compared to only 18-25% in the second period. However, D_{max} was almost equal or lower (-3 to 13 Gy) in the second time period. We also found a larger relative variation in dose in the second period versus the first time period. The uniformity of the regimens in the first period contra the differences in the regimens of the second time period was the main reason to changes in the variation in doses. During the second period, high doses were administered only to a small part of the heart, with a steep dose gradient to the other parts due to the usage of MVT. Electron beams and tangential photon beams were used in the second period. Both beams result in a small dose contribution to the heart due to the limited dose range and technique. Most of the five volumes of interest in CT set L had lower or almost equal dose than the other CT sets (Table S4). One reason for this is the distance between the heart and skin which is larger for CT set L than the other sets (Figure S1).

Uncertainty of estimated doses

A number of factors contribute to the uncertainty of the estimated dose in the different volumes. Some of the factors are calculation of the dose, reconstruction of the unknown anatomy, and uncertainty of a given dose. In addition, the uncertainty of the dose in the different volumes varies due to the location of the volume relative to the border of field, shape of the patient, size of the volume of interest, treatment technique, and dose parameters. The calculation by the computer of the dose in the body is expected to be less uncertain for MVT photon beams than OVT beams. The mean dose in large volumes compared to small volumes are less uncertain

mainly due to the location relative to the field and dose gradients. However, the uncertainty in D_{max} is almost independent of the volume of the organ but strongly related to distance to the penumbra of the fields. Thus, in this investigation, the largest uncertainty is found, in the coronary arteries with OVT. A rough estimation of the presented dose in most women, should be within ±15%. The uncertainty in the dose can be larger when the organ is located in the penumbra region. One example is for LAD with tangential MVT photon beams. The smallest uncertainty is found in the presented mean dose in heart and LV for OVT and MVT and within ±10% for most of the women.

References:

1. Landberg T, Chavaudra J, Dobbs J, Gerard JP, Hanks G, Horiot JC, et al. 2. Volumes. Reports of the International Commission on Radiation Units and Measurements. 1999 1999/11/01;os-32(1):3-20.
2. Taylor CW, Nisbet A, McGale P, Goldman U, Darby SC, Hall P, et al. Cardiac doses from Swedish breast cancer radiotherapy since the 1950s. Radiother Oncol. 2009 Jan;90(1):127-35. PubMed PMID: 19008005. Epub 2008/11/15. eng.
3. Amols HI, Lagueux B, Cagna D. Radiobiological effectiveness (RBE) of megavoltage X-ray and electron beams in radiotherapy. Radiat Res. 1986 Jan;105(1):58-67. PubMed PMID: 3080801. Epub 1986/01/01.

Structure/ CT set	Volume (cm3)		
	M	S	L
Whole heart	627	551	727
LAD	10	14	14
RCA	9	9	11
LCA	3	4	4
Left ventricle	192	148	192

Table S1. Volumes of the different structures by CT set: M=medium, S=small, L=large.

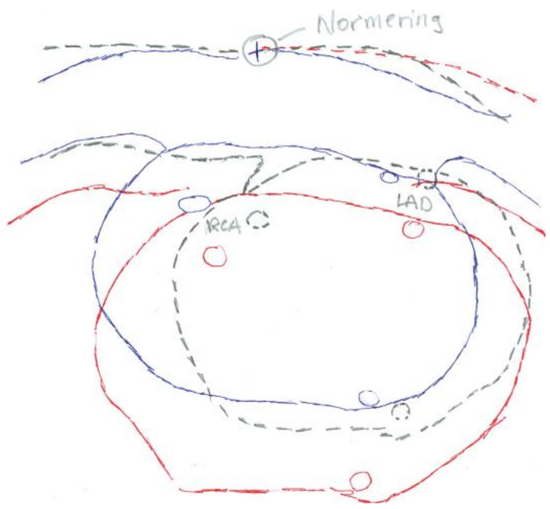

Figure S1. Heart and skin contours on a central CT slice from each CT set normalized to the midline of the CT sets L (red, large), S (dotted, small) and M (blue, medium).

IMC target and postop. treatment	Chest wall target and postop. treatment	Other target and intention	Treated side	
Beam / fields / dose per fx/dose	Beam / field / dose per fx / dose	Beam / target / dose per fx/ dose	Right	Left
OVT / 3 / 29-33	OVT / oblique / 3 / 26-33		31	59
OVT / AP / 3 / 26-33	OVT / oblique / 3 / 13-29	OVT / preop breast / Tangent 3 / 21-26	7	6
OVT / AP / 3-4 / 33-40	OVT / oblique / 3-4 / 33-40	OVT / palliative spine / PA field / 4 / 30-40	2	
OVT / AP-PA or AP / 3 / 26-36	OVT / AP-PA or oblique / 3 / 26-36		2	1
Electrons / AP / 2-4 / 40-55	Electrons / oblique / 2-4 / 40-67		3	4
Electrons / AP / 4-5 / 35-45	Photons / tangent / 5,0 / 45-50		2	4
Electrons / AP / 4 / 36		Photons / intact breast / Tangent 4 / 68		1
Electrons / AP / 4-5 / 40-50			7	4
		Photons / postop. breast / Tangent / 2 / 48-52	4	7
	Photons / tangent / 5 / 45		2	
		Photons / intact breast / Tangent / 2 / 76	1	
Photons / AP / 4 / 45				2
OVT/ AP / 4 / 44	Photons / tangent / 4 / 48			3
OVT+photons / AP / 5 / 45			1	2
Electrons / AP / 2-4 / 40-68			Two bilateral IMC fields / 4	
OVT / AP / 3-7 / 28-41			Two bilateral IMC fields / 3	
OVT / AP / 3 / 29-33	OVT / oblique / 3 / 21-33		Two bilateral chest wall fields / 2	
Mixed beams / AP / 2-5 / 36-56	Mixed beams / oblique / 2-5 / 21-50		Other bilateral combinations / 4	

Table S2. Beam qualities, treatment techniques and prescribed doses for OVT at the skin surface, MVT electron beams at a specific depth, and MVT with tangential photon beams at the middle of the breast.

A

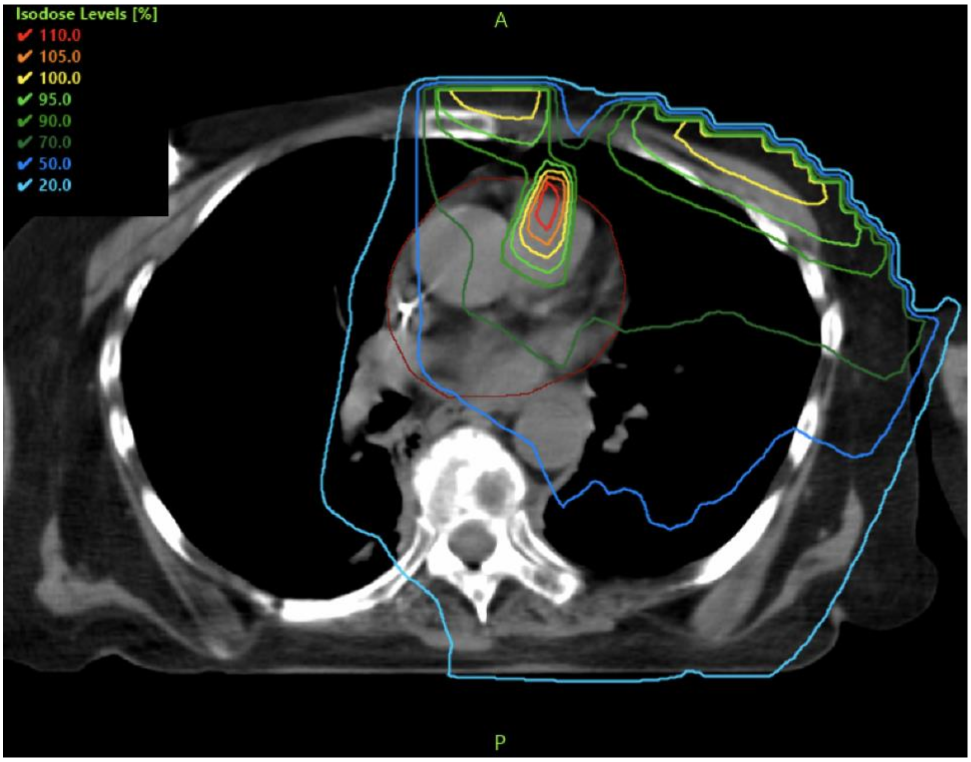

B

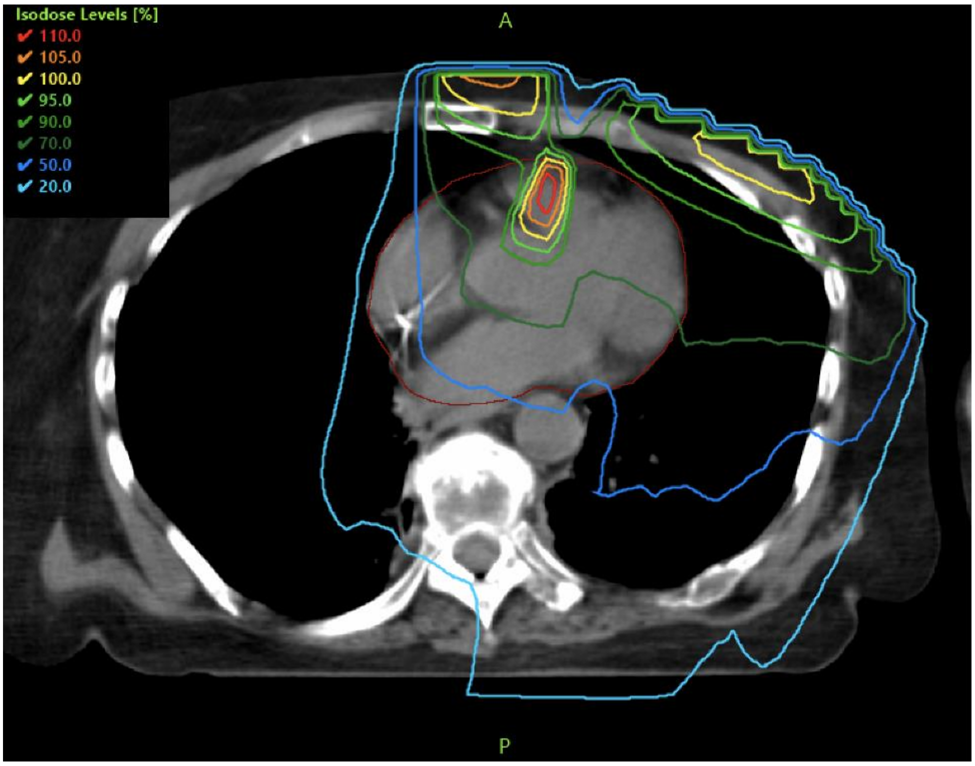

Figure S2. Transverse sections proximal (A) and mid-heart (B). Dose estimations with isodose curves from reconstruction of left-sided treatment corresponding to the most common regimen among patients exposed to RT (OVT targeting IMC and chest wall).

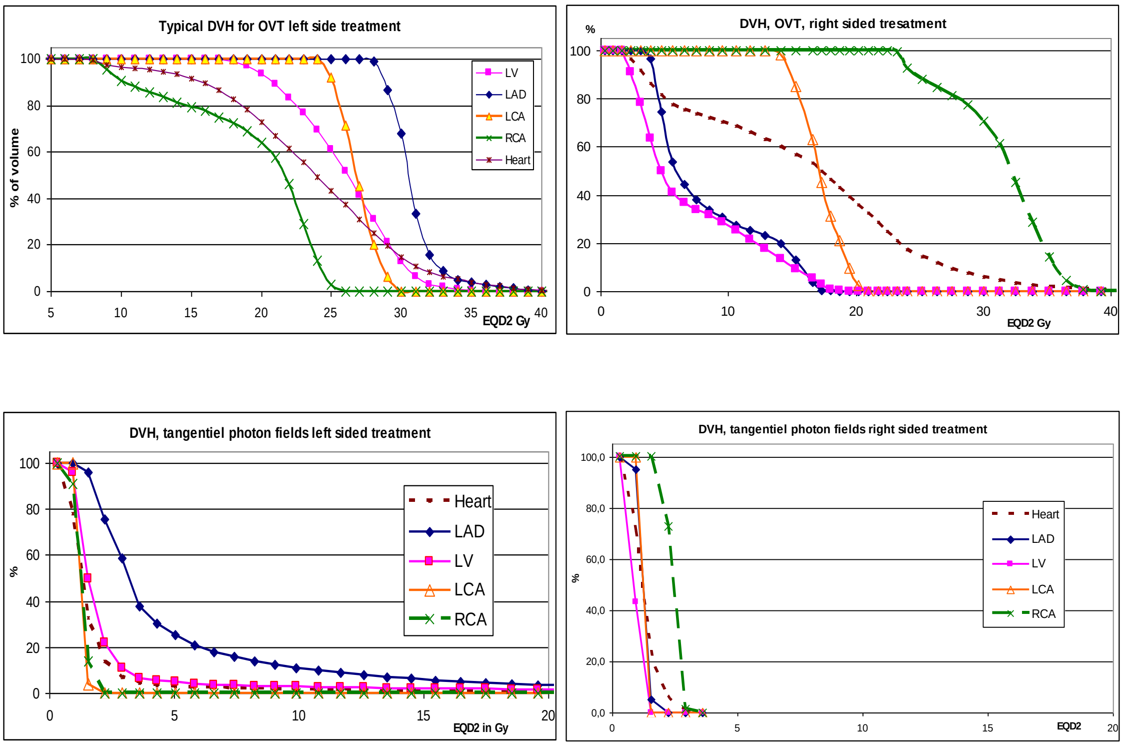

Figure S3. Four DVHs of two typical treatment techniques. Top OVT. Bottom, tangential MVT techniques. Left, left-sided treatment. Right, right-sided treatment.

Volume	Average of mean EQD2	Average of near maximum EQD2
Whole heart	12±6 (1,28)	30±11 (2,86)
LAD	15±12 (0,77)	23±16 (0,94)
RCA	17±9 (1,47)	24±11 (1,59)
LCA	13±8 (0,31)	15±9 (1,37)
Left ventricle	10±8 (0,32)	19±12 (1,60).

Table S3. The average, standard deviation, and range of the mean and near maximum of equivalent dose in 2 Gy fractions with α/β = 3 Gy (EQD2) for the 168 patients and three CT sets.

	Average of mean EQD2 Gy±1 standard deviation.						Average of near maximum EQD2 Gy±1 standard deviation					
	Left-sided treatment			Right-sided treatment			Left-sided treatment			Right-sided treatment		
CT set	M	S	L	M	S	L	M	S	L	M	S	L
Heart	15±5	17±7	11±5	11±5	8±4	7±4	34±11	35±8	25±7	32±12	26±10	21±8
LAD	26±10	24±7	18±7	4±3	2±2	3±3	35±14	35±10	23±7	11±7	5±4	8±6
RCA	11±6	18±8	12±7	26±9	17±7	18±9	18±8	26±9	18±9	34±12	24±9	25±9
LCA	18±8	17±8	14±7	10±7	5±4	8±5	20±8	20±8	16±7	12±8	9±6	10±6
LV	16±6	17±8	12±6	4±3	2±1	3±2	31±8	29±7	19±8	12±8	6±4	8±3

Table S4. Cardiac doses with 1standard deviation for 155* subjects. Volumes are divided into left- and right-sided treatments and the three CT sets (small, medium, and large).
*bilateral treatments (13 patients) not included.

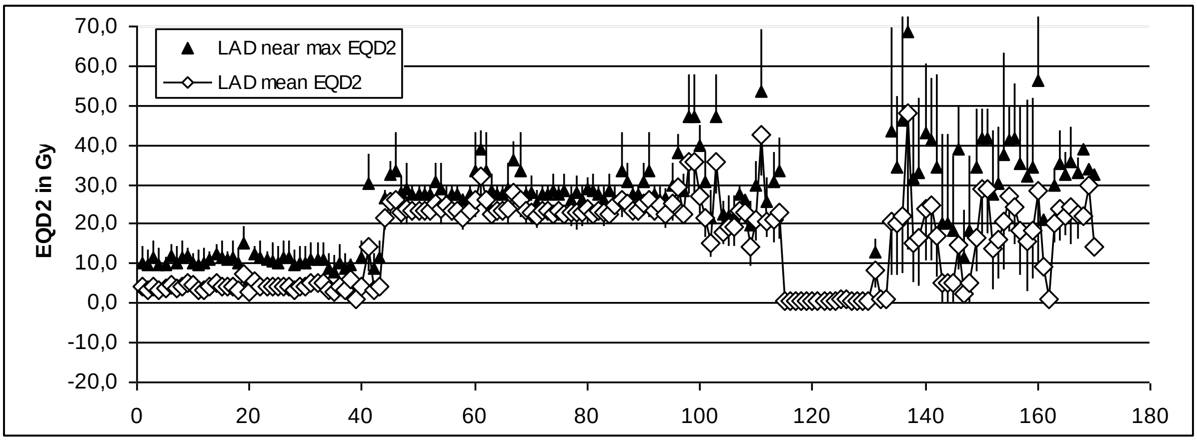

LAD near max EQD2
LAD mean EQD2
EQD2 in Gy

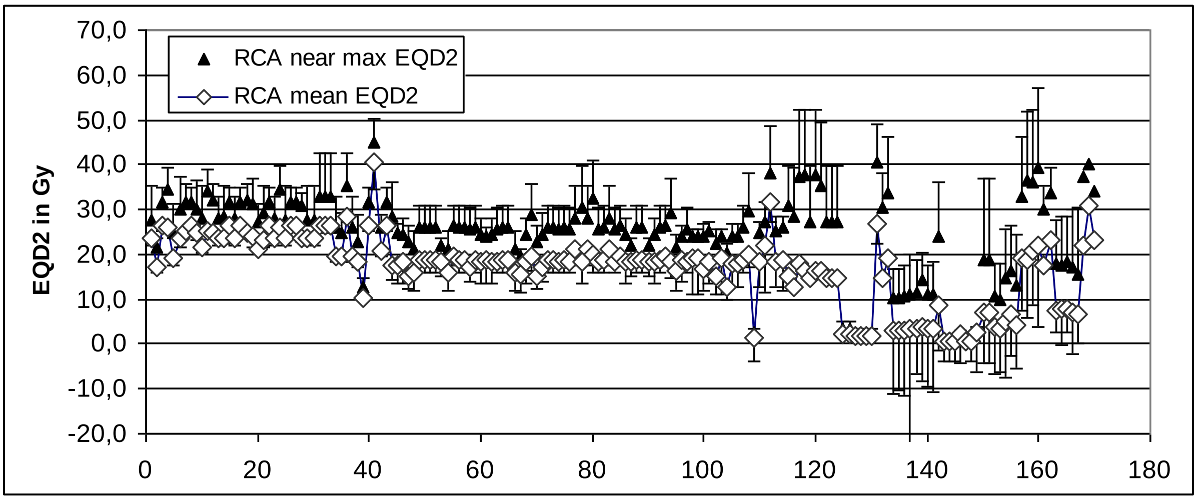

RCA near max EQD2
RCA mean EQD2
EQD2 in Gy

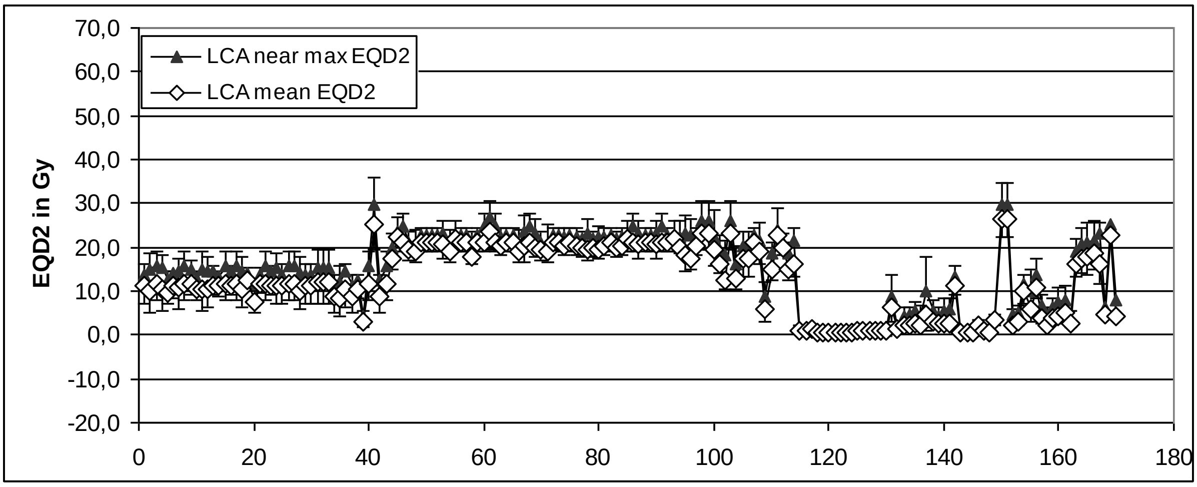

LCA near max EQD2
LCA mean EQD2
EQD2 in Gy

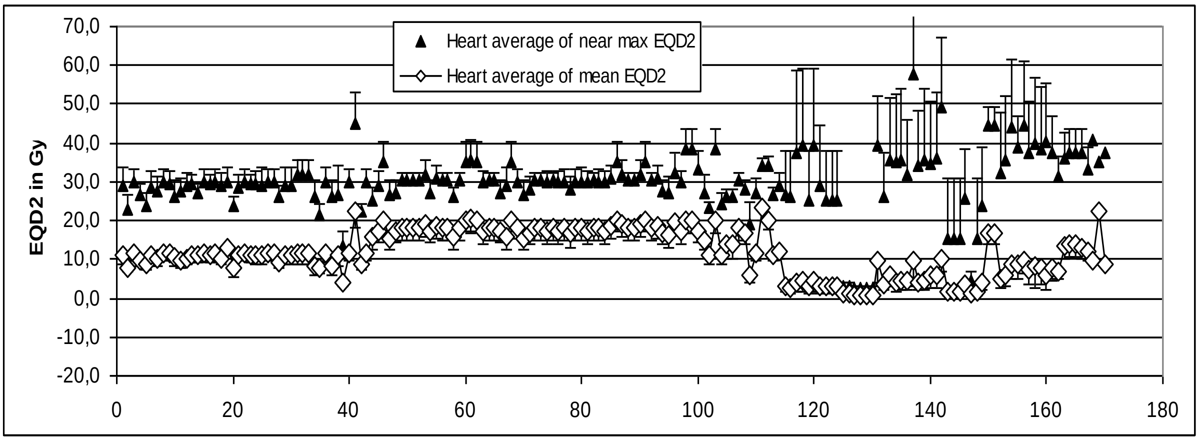

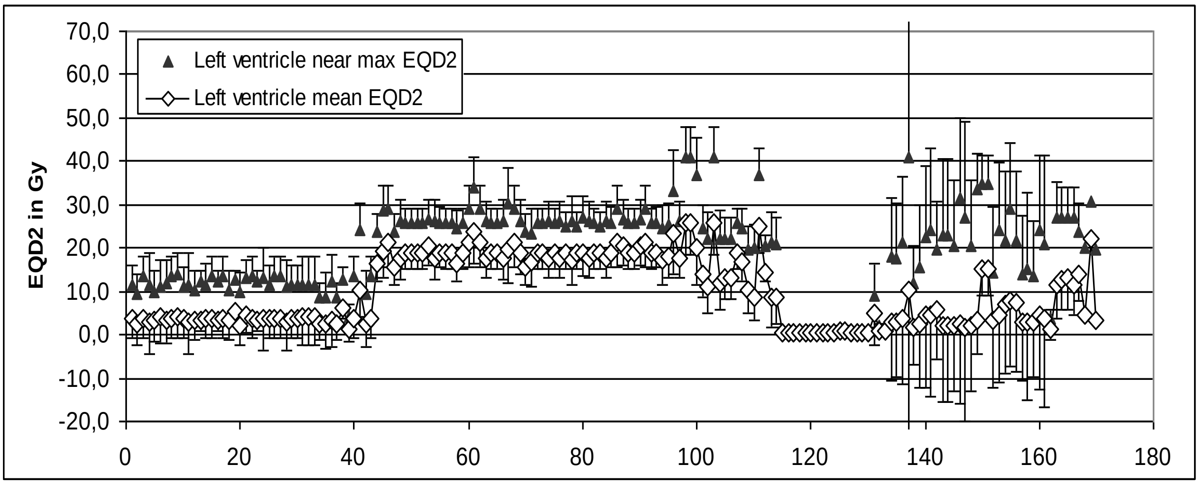

Figure S4. Average of the three CT sets of mean and near maximum EQD2. Patients listed on the vertical axis with dose standard deviation in one direction only: 1-42 OVT right-sided treatments, 43-108 OVT left-sided treatments, 109-113 OVT bilateral treatments, 114-123 electron beam right-sided treatments, 124-130 photon beam right-sided treatments, 131-132 electron and photon beam right- sided treatments, 133-140 electron beam left-sided treatments, 141-149 photon beam left-sided treatments, 150-154 electron and photon beam left-sided treatments, 155-159 electron beam bilateral treatments, 160-168 mixed beam quality.
